# Supplementary material for: Vitamin D Modified DSS-Induced Colitis in Mice via STING Signaling Pathway
Source: Biology (Basel). 2025 Jun 18;14(6):715. doi: 10.3390/biology14060715 (PMC12190092; doi:10.3390/biology14060715)
Supplement: Supplementary file 1 [file biology-14-00715-s001.zip › Supplementary Materials S1: 16s-report of gut microbiota/Groups/diff_analysis/heatmap/GBMclusterdiffheatmap.pdf]

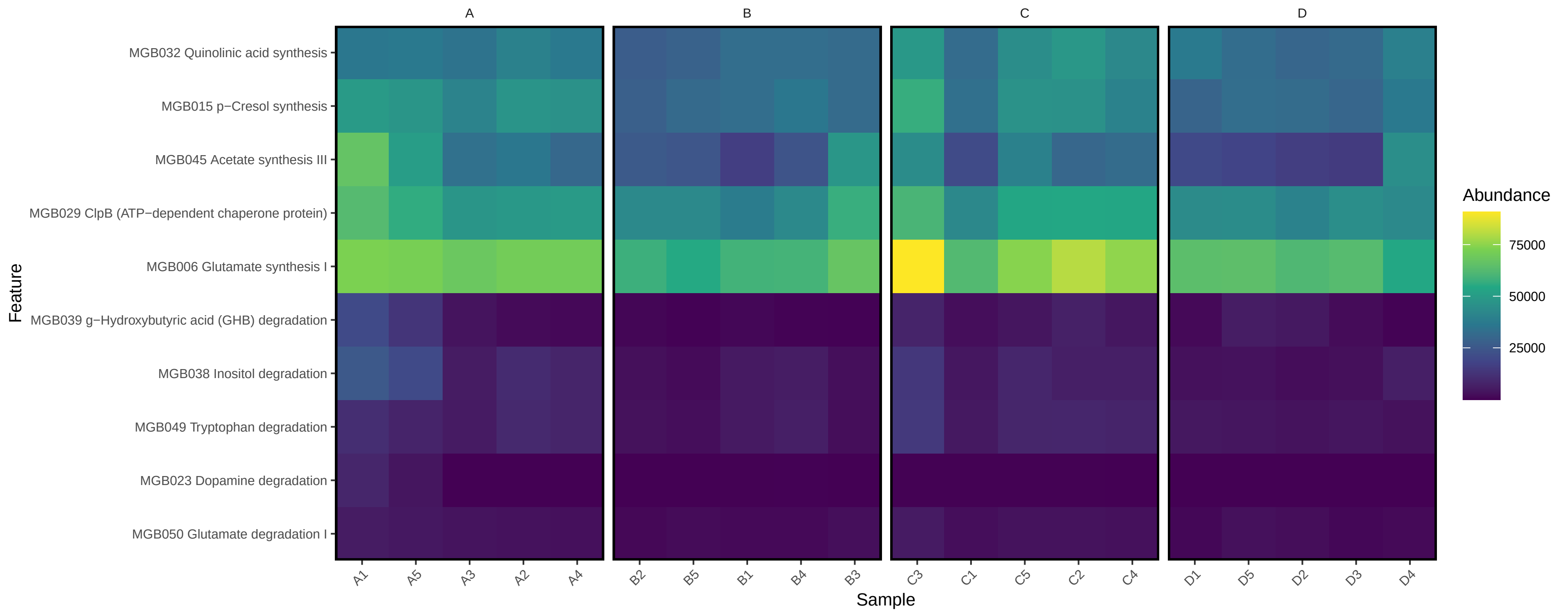
A
B
C
D
MGB032 Quinolinic acid synthesis
MGB015 p−Cresol synthesis
MGB045 Acetate synthesis III
MGB029 ClpB (ATP−dependent chaperone protein)
MGB006 Glutamate synthesis I
MGB039 g−Hydroxybutyric acid (GHB) degradation
MGB038 Inositol degradation
MGB049 Tryptophan degradation
MGB023 Dopamine degradation
MGB050 Glutamate degradation I
Feature
A1
A5
A3
A2
A4
B2
B5
B1
B4
B3
C3
C1
C5
C2
C4
D1
D5
D2
D3
D4
Sample
Abundance
75000
50000
25000
